# Reperfusion therapy for acute ischemic stroke: where are we in 2023?

**DOI:** 10.1055/s-0043-1777721

**Published:** 2023-12-29

**Authors:** Sheila Cristina Ouriques Martins, Octávio Marques Pontes-Neto, Arthur Pille, Thaís Leite Secchi, Maramélia Araújo de Miranda Alves, Letícia Costa Rebello, Jamary Oliveira-Filho, Marcos Christiano Lange, Gabriel R. de Freitas, João Brainer Clares de Andrade, Letícia Januzi de Almeida Rocha, Daniel da Cruz Bezerra, Ana Claudia de Souza, Leonardo Augusto Carbonera, Raul Gomes Nogueira, Gisele Sampaio Silva

**Affiliations:** 1 Universidade Federal do Rio Grande do Sul, Hospital de Clínicas de Porto Alegre, Porto Alegre RS, Brazil.; 2 Hospital Moinhos de Vento, Porto Alegre RS, Brazil.; 3 Universidade de São Paulo, Faculdade de Medicina de Ribeirão Preto, Ribeirão Preto SP, Brazil.; 4 Universidade Federal de São Paulo, Hospital São Paulo, São Paulo SP, Brazil.; 5 Hospital de Base de Brasília, Brasília DF, Brazil.; 6 Universidade Federal da Bahia, Salvador BA, Brazil.; 7 Universidade Federal do Paraná, Hospital de Clínicas, Curitiba PR, Brazil.; 8 Hospital Quinta D'Or, Rio de Janeiro RJ, Brazil.; 9 Hospital Universitário Professor Alberto Antunes, Maceió AL, Brazil.; 10 Hospital Pró-Cardíaco, Rio de Janeiro RJ, Brazil.; 11 University of Pittsburgh, Pittsburgh PA, United States.

**Keywords:** Stroke, Ischemic Stroke, Reperfusion, Thrombolytic Therapy, Thrombectomy, Acidente Vascular Cerebral, Acidente Vascular Cerebral Agudo, Reperfusão, Terapia Trombolítica, Trombectomia

## Abstract

Over the last three decades, stroke care has undergone significant transformations mainly driven by the introduction of reperfusion therapy and the organization of systems of care. Patients receiving treatment through a well-structured stroke service have a much higher chance of favorable outcomes, thereby decreasing both disability and mortality. In this article, we reviewed the scientific evidence for stroke reperfusion therapy, including thrombolysis and thrombectomy, and its implementation in the public health system in Brazil.

## Resumo

Nas últimas três décadas, o tratamento do AVC sofreu transformações significativas, impulsionadas principalmente pela introdução das terapias de reperfusão e pela organização dos serviços de AVC. Os pacientes que recebem tratamento em um serviço de AVC bem estruturado têm uma probabilidade muito maior de resultados favoráveis, diminuindo assim a incapacidade funcional e a mortalidade. Neste artigo, revisamos as evidências científicas para as terapias de reperfusão do AVC, incluindo trombólise e trombectomia e sua implementação no sistema público de saúde no Brasil.

## INTRODUCTION

Stroke is the second leading cause of death and the leading cause of disability worldwide. Annually, more than 12 million people are affected by stroke, and about 6.5 million die due to the disease. 1 In Brazil, 400,000 strokes occur each year. The lowest-income population is the most affected, and 80% of stroke patients require care in the public health system, called SUS (Unified Health System). 2 3 The disability generated by stroke significantly impacts society, is highly associated with functional dependence, the need to increase the use of health resources and generates a heavy economic impact.

For more than 30 years, stroke was the first cause of death in Brazil, 2 3 becoming the second in 2011 due to the organization of acute stroke care, 3 an effective and cost-effective way to reduce mortality and disability in stroke. Unfortunately, after the COVID-19 pandemic, stroke became again the first cause of death in the country.

It is necessary to optimize urgent care through a coordinated stroke pathway for rapid recognition and treatment of patients using evidence-based protocols. 4 According to national and international clinical guidelines, 5 6 7 8 the interventions with a high level of evidence of benefit in acute stroke care are acetylsalicylic acid in the first 24 hours of ischemic stroke for non-thrombolysed patients to avoid stroke recurrence; admission of all patients to stroke units, with a multidisciplinary team trained and engaged in early assessment and rehabilitation protocols; intravenous (IV) thrombolytic treatment in disabling ischemic stroke within 4.5 hours of symptom onset; mechanical thrombectomy (MT) in stroke with large vessel occlusion (LVO) within 24 hours of symptom onset, and decompressive craniectomy of malignant middle cerebral artery (MCA) infarction within 48 hours. The first four out of these five recommendations have already been implemented in the Brazilian SUS. 2 8 9 10 Mechanical thrombectomy was approved by the Ministry of Health on February 2021, 11 and is currently awaiting promulgation of the final ordinance for its effective implementation.

Since 1995, with the accumulation of evidence on the efficacy of thrombolysis in stroke, its care has become a medical emergency that requires immediate treatment, and there is a strong temporal dependence between treatment and the development of long-term functional disability. 12 13 Since then, hospitals worldwide have been organized with well-defined protocols, care pathways, and well-trained medical and nursing teams at each stage of the process, allowing for rapid evaluation and treatment initiation.

In this article, we reviewed the scientific evidence of stroke reperfusion therapy, including thrombolysis 12 13 and thrombectomy, 14 15 16 17 18 19 20 and its implementation in the public health system in Brazil.

## METHODS

We searched the Cochrane Library, MEDLINE, Embase, LILACS, and SciELO for articles published in any language between January 1, 1995, and October 15, 2023. We used the search terms "ischaemic", "ischemic stroke", "thrombolysis", "reperfusion", "recanalization”, “endovascular thrombectomy/mechanical thrombectomy/thrombectomy", "large vessel occlusion" and "clinical trial" or "meta-analysis". We also searched the references of articles identified by this search strategy and selected those relevant.

Also, we described the main milestones in the implementation of reperfusion therapy in Brazil.

## OVERVIEW OF THE TREATMENT

Currently, the main reperfusion strategies evidence-based for patients with acute ischemic stroke (AIS) are IV thrombolysis and MT. Rapid reperfusion of the compromised vessel in a stroke can limit the ischemic area and decrease or even reverse disability. Therefore, fast diagnosis and initiation of treatment are the most critical aspects of management in a safe and monitored environment with trained staff.

### Measures to restore cerebral blood flow: intravenous thrombolysis

Thrombolytic treatment aims to dissolve the thrombus and restore regional blood flow. Recombinant tissue plasminogen activator (tPA) is the only thrombolytic approved for this purpose so far 5 6 with current promising studies also demonstrating the benefit of tenecteplase (TNK), which will probably be added to the guidelines in the near future.

#### *Clinical trials*

The use of tPA in stroke was approved by the Food and Drug Administration (FDA) after the study conducted by the National Institute of Neurological Disorders and Stroke (NINDS) 12 in American hospitals, a randomized clinical trial (RCT), double-blind, controlled. This trial included 624 stroke patients treated with tPA 0.9mg/kg or placebo within three hours of symptom onset. The tPA group had 30% more patients with minimal or no neurological deficit measured by the National Institute of Health Stroke Scale (NIHSS) of 0 or 1 point, (NIHSS) three months after stroke. There was a higher rate of symptomatic intracerebral hemorrhage (SICH) in the tPA group (6.4% x 0.6% p < 0.001) but no increase in mortality (17% tPA group vs. 21% placebo). The more severe the initial neurological deficit and the degree and extent of hypodensity on CT, the greater the risk of SICH. The benefit was demonstrated in all stroke subtypes. 12 13 14 15 16 17 18 19 20 21

**Figure 1:**
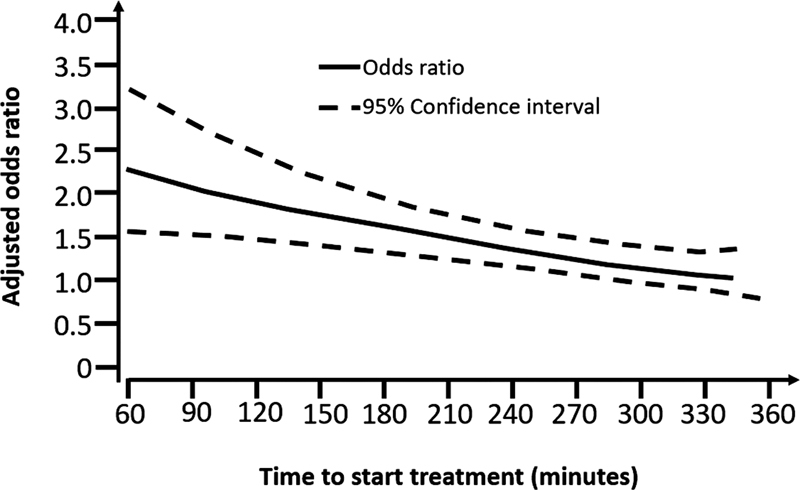
Odds ratio for favorable prognosis at three months in patients treated with tPA compared with controls by the time of initiation of treatment. (Adapted from Lancet 2004 13 ).

Three other large RCT evaluated IV tPA in AIS: the European Cooperative Acute Stroke Study (ECASS I and II) and Alteplase Thrombolysis for Acute Noninterventional Therapy in Ischemic Stroke (ATLANTIS). These studies used different doses of tPA or other treatment windows that failed to show a benefit. A meta-analysis of the NINDS, ECASS I and II, and ATLANTIS studies (2775 patients) published in 2004 13 studying patients treated between 0-6h showed that the earlier the administration of tPA, the better the outcomes ( Figure 1 ). The group treated within 90min was 2.8 times more likely to have minimal or no disability at three months, with a Number Needed to Treat (NNT) of 7; treatment between 181-270min led to an odds ratio (OR) of 1.4 and no benefit was observed among patients treated between 271-360min. The SICH in the tPA group was 5.9% vs. 1.1% in the placebo group (p < 0.0001). In 2008, the benefit of using IV tPA up to 4.5h was confirmed in the ECASS III clinical trial, 22 extending the therapeutic window.

The IST-3 (Third International Stroke Trial) 23 evaluated patients with stroke symptoms up to 6h of symptoms to receive IV tPA vs placebo selected by non-contrast head CT (NCCT). The study included a population outside the formal indication for treatment, and of the 3035 patients in the study, 1617 (53%) were over 80 years of age. At six months, the mortality was similar between groups (26.9% tPA vs 26.7% non-tPA). There was an increased risk of fatal hemorrhagic transformation with a tPA of 5.8%, as already demonstrated in previous studies. Functional independence was not significantly different between the groups (37% versus 35%). Therefore, there was no benefit to using tPA in stroke until 6h from the symptoms, but the effect of treatment in those >80 years was at least as good as in those ≤80 years, showing that the treatment can be used in these patients in the previously defined time window.

A systematic review with meta-analysis 24 gathered 12 studies and evaluated 7012 patients who used tPA versus conventional treatment up to six hours of stroke onset. There was no difference in mortality between groups, but there was an Absolute Risk Reduction in the composite outcome of death and severe functional disability of 4% (ARR = 4%, 95% CI 1.7-6%), with an NNT of 25. The benefits were more pronounced in patients treated up to 3h, with an NNT of 11 to achieve an mRS0-2 (ARR = 0.9%; 95% CI 0.46-1.34) and an NNT of 19 to obtain mRS0-1 (ARR = 5.4%; 95% CI 3.2-7.6). Patients thrombolysed within 3h (>80 years or ≤ 80 years), had higher survival and/or independence when compared to placebo-treated patients, NNT = 5 (ARR = 20.7%, 95% CI 14.4-27.0). Despite being a subgroup evaluation, the statistical power was 98%. The exact same population described, but thrombolyzed within 6h, achieved the benefits of survival and/or independence, NNT = 4 (ARR = 25.3%, 95% CI 21.8-28.8). The IST-3 Study demonstrated that patients with NIHSS > 25 also benefit from IV thrombolysis.

In 2016, the ENCHANTED study 25 randomized 3310 patients to receive either the usual dose of IV alteplase (0.9mg/kg, 10% bolus and the remainder infusion within one hour) or the low dose (0.6mg/kg, 15% bolus, and the remainder infusion within one hour) in patients treated within 4.5h of symptom onset. The primary outcome, minimal or no disability at 90 days (mRS0-1) and occurred in 53% of the low-dose-group and in 51% in the standard-dose group, not reaching non-inferiority. Low dose was not inferior in the ordinal analysis of the mRS scores (ability to improve one category of the mRS score). SICH occurred in 1.0% of the low-dose and in 2.1% in the standard-dose group (p = 0.01); fatal events within seven days occurred in 0.5% and 1.5%, respectively (p = 0.01). In practice, we maintain the standard dose of 0.9 mg/kg, and many services use the low dose for patients with a potentially higher risk of bleeding.

#### *Intravenous thrombolysis in extended time window*

In recent years, evidence has accumulated regarding the potential benefits of IV thrombolysis beyond the traditional 4.5h window for AIS. Two pivotal trials, EXTEND 26 and WAKE-UP, 27 have contributed significantly to this discourse. The EXTEND demonstrated that patients with salvageable brain tissue identified via perfusion imaging could benefit from IV thrombolysis administration up to 9 hours from symptom onset. On the other hand, the WAKE-UP trial focused on patients who woke up with stroke symptoms, making the time of onset uncertain. Using MRI criteria to identify patients with recent ischemia (diffusion-flair mismatch), the study revealed that this subset of patients also benefited from IV thrombolysis compared to placebo. These trials underscore the potential of imaging-guided thrombolysis in broadening the therapeutic window, though patient selection remains paramount.

#### *Symptomatic intracerebral cerebral hemorrhage*

The independent factors related to the risk of SICH after the use of tPA in patients with AIS are hypodensity on NCCT > 1/3 of the territory of the MCA (OR 9.38), presence of mass effect on CT up to 3h, even if involved territory is less than 1/3 of the MCA (8-fold increase the risk), age > 75 years, BP > 180/105mmHg at infusion initiation, diabetes (OR 2.69) and NIHSS > 20 (17% chance of intracranial bleeding compared to 3% in patients with NIHSS < 10). 28 29 30 Despite the higher risk of bleeding, there is no upper age limit for treatment, and the elderly should not be excluded on the basis of age alone. The same recommendation is made for patients with elevated NIHSS, who should be treated if the early ischemic area is not extensive.

### Measures to restore cerebral blood flow: mechanical thrombectomy

**Figure 2:**
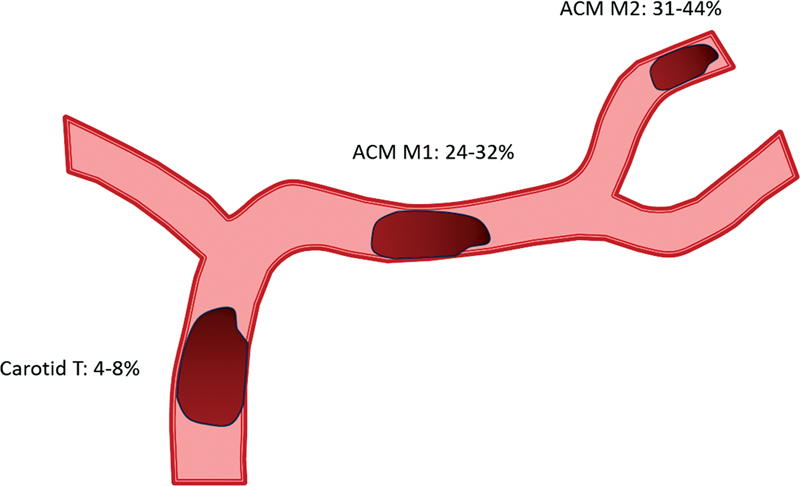
Recanalization rates with tPA by affected segment.

Despite the indisputable benefit of tPA, there is still a considerable portion of patients (25-30%) with LVO (intracranial internal carotid artery-ICA, proximal MCA and basilar artery), who had very low recanalization rates ( Figure 2 ) 31 and high rates of mortality and disability when treated only with tPA, with good functional outcomes being achieved by only 25% of patients, 32 making clear the need for a new therapeutic modality.

#### *Neutral studies*

In 2013, three RCT reported neutral results (Synthesis, 33 IMS-III 34 and MR-RESCUE 35 ) comparing the best medical treatment (BMT) with MT, almost leading to the erroneous conclusion that MT had no place in the management of AIS. The main criticisms of these studies were: long inclusion period due to difficulty in recruiting patients, small number of patients per center (low volume and experience), absence of vascular imaging to detect the site occlusion, no evaluation of tissue viability, heterogeneity of the techniques used to perform the procedures with first-generation devices or intra-arterial thrombolytics only. The analysis of these studies clearly showed that the selection of patients and the therapeutic methods evaluated were key elements that should be addressed in the design of future studies.

#### *New evidence – positive studies*

The scenario of endovascular therapy in AIS changed radically after the sequential publications of 5 pivotal studies 14 15 16 17 18 testing a new generation of stent retriever devices (December 2014-April 2015), which showed the consistently clear superiority of MT over the BMT in reducing disability at 90 days in patients with AIS due to LVO of the anterior circulation. The first study published was the Multicenter Randomized Clinical Trial of Endovascular Treatment for Acute Ischemic Stroke (MR-CLEAN) 14 in the Netherlands, with subsequent studies all stopped early due to efficacy and loss of equipoise.

#### *Study design and selection criteria*

All studies consisted of RCT, which focused on the selection of patients with confirmed occlusion in the intracranial ICA or proximal MCA (by CT angiography-CTA, MR angiography-MRA- or digital subtraction angiography), use of second-generation stent retrievers (Solitaire ^®^ ) and time from symptoms onset to randomization 6-12 hours. Extending the Time for Thrombolysis in Emergency Neurological Deficits with Intra-Arterial Therapy (EXTEND-IA) 17 used CT perfusion (CTP) imaging to include patients according to mismatch profile (difference between potentially salvageable tissue and non-viable tissue). ESCAPE 15 study used good collateral circulation as an inclusion criterion. The MR-CLEAN 14 study had the most inclusive criteria, as it did not use the extension of early infarcted area on NCCT to exclude patients.

#### *Clinical trials and meta-analyses – anterior circulation*

The MR-CLEAN 14 showed functional independence (mRS0-2) in 32.6% of MT group vs. 19.1% in BMT group, (p < 0.01, aOR 2.16, 95% CI 1.39-3.38), NNT of 8. The mortality was 18.4%, and SICH 7.7%, without difference between the groups.

The ESCAPE trial 15 in Canada was stopped early because of the results of the MR-CLEAN. It demonstrated an mRS≤2 in 53% MT group versus 29.3% BMT group (p < 0.001). The adjusted OR was 3.1, 95% CI 2.0-4.7, with the ordinal analysis favoring the MT group in the 90 days (p <0.001). The NNT was 4, with a mortality of 10.4 and 19.0% between the MT and BMT group (p = 0.04). SICH was 3.6% in the MT group, with no significant difference.

In Australia, EXTEND-IA 17 was terminated after randomization of only 70 patients. The OR was 2.0 for the ordinal analysis (95% CI 1.2-3.8; p = 0.006) in favor of thrombectomy, with NNT of 2.8. The proportion of patients who achieved an mRS≤2 in the MT group was 71% versus 40% in the BMT group (OR 4.2, 95%CI 1.4-12; p = 0.010). In MT group, no SICH was observed and mortality was 9%, similar to the control arm.

Solitaire with the Intention for Thrombectomy as Primary Treatment for Acute Ischemic SWIFT-PRIME 16 study was conducted in the United States and Europe. The study was halted by the Data and Safety Monitoring Board (DSMB) after an interim analysis due to the superiority of the MT group. The study recruited 196 patients, and the MT group achieved mRS0-2 in 60% compared to 35% of the BMT group (aOR 2.03, 95% CI 1.36-3.03; p < 0.001). The NNT was 4 to achieve functional independence and 2.6 to improve one point on the mRS. There was no statistical difference in SICH or mortality.

In Spain, the Endovascular Revascularization with Solitaire Device versus Best Medical Therapy in Anterior Circulation Stroke within 8h (REVASCAT) 18 was discontinued early, shortly after the publications of ESCAPE, EXTEND-IA and MR-CLEAN, because they resulted in a loss of equipoise. In the ordinal analysis of the mRS, the adjusted OR was 1.7, 95%CI 1.05-2.8) in favor of the intervention. The proportion of patients who became functionally independent was 43.7% in the MT group versus 28.2% in the BMT group (aOR 2.1, 95% CI 1.1-4.0). The NNT was 6.5 to prevent disability or death. No statistically significant difference was found in mortality or SICH.

The metanalysis Highly Effective Reperfusion Evaluated in Multiple Endovascular Stroke Trials (HERMES) 36 analyzed data from 1,287 individual patients of these five studies and clarified the treatment effect of MT in important clinical and radiological subgroups. HERMES showed that at 90 days functional independence was achieved in 46.0% in MT and 26.5% in the BMT group, with NNT of 2.6 in ordinal analysis and similar rates of tPA administration and SICH in both groups. The benefit remained consistent across subgroups of patients, such as those who did not receive tPA, including being slightly higher in those over 80 years of age. The severity of the event, measured by the NIHSS scale, was a prognostic factor but not a modifier of the treatment effect.

**Figure 3:**
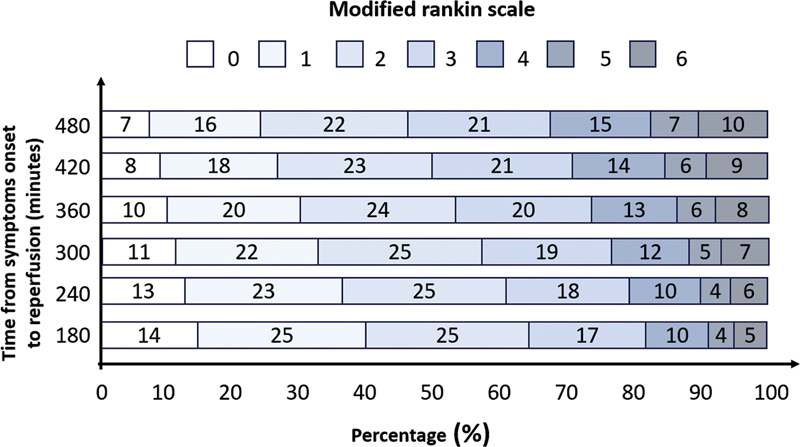
Relationship between time from onset of symptoms in treatment and functional outcome (adapted from Saver, JAMA 2016 35 ).

**Figure 4:**
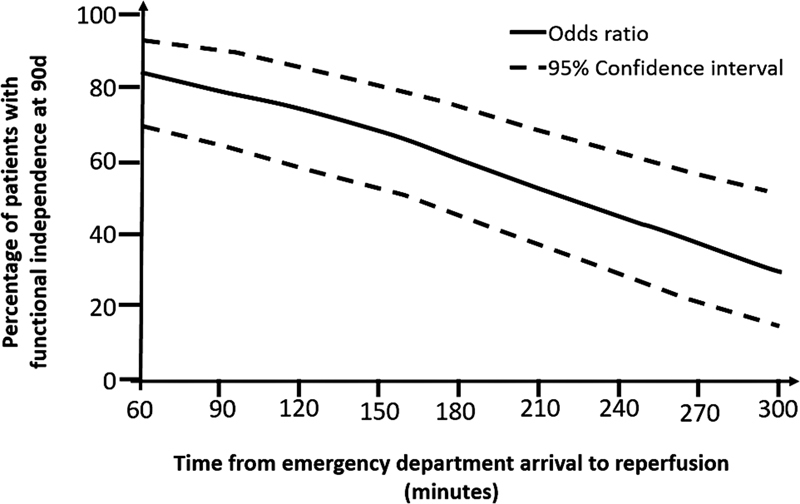
Relationship between door-reperfusion time and functional outcome (adapted from Saver, JAMA 2016 35 ).

A second meta-analysis 37 of the same studies demonstrated that time to successful reperfusion is highly associated with functional outcome, with good results when thrombectomy was performed up to 7.3h after symptoms, with clearly more significant benefit with earlier interventions (<3h) ( Figure 3 ). For every 4 minutes lost from arrival to reperfusion, 1 in 100 patients had worse functional outcome ( Figure 4 ). There was a lower loss of benefit (for the same time elapsed until reperfusion) in patients with higher infarction volume (core) on arrival (ASPECTS 7-8) compared to minors (ASPECTS 9-10), reaffirming that speed in performing MT is the key to achieving the best results.

#### *Thrombectomy trial in Brazil*

In 2015, despite the five published RCT showing the benefit of MT in LVO-related ischemic stroke, none of them were performed in low and middle-income countries (LMIC). This led the Brazilian government to sponsor an RCT, to assess the efficacy and cost-effectiveness of this therapy in our country, and if the treatment should be incorporated into the Universal Public Health Care System. The Randomization of Endovascular Treatment with Stent-retriever and/or Thromboaspiration versus Best Medical Therapy in AIS due to LVO Trial (RESILIENT) 38 was to determine the safety and efficacy of MT in the public health of a developing country. The initial sample size was calculated at 690 individuals, but in the first interim analysis of 174 patients, the DSMB discontinued the study due to efficacy favoring the MT group. The final results of 221 patients showed that the OR for a better distribution of scores on the mRS at 90 days was 2.28 (95% CI, 1.41-3.69; p = 0.001), favoring thrombectomy. The percentage of patients with mRS0-2 was 35.1% in the MT group and 20.0% in the BMT (difference, 15.1%; 95% CI, 2.6-27.6), and the NNT was 6.6. SICH occurred in 4.5% of patients in each group. These results showed for the first time the feasibility, safety, and efficacy of MT in acute stroke in low-resource settings demonstrating that the treatment can be implemented in many more countries globally. Also, a cost-effectiveness study was performed 39 showing that the treatment in public health in Brazil is highly cost-effective.

#### *Time window expansion – tissue window concept*

Two RCTs – DAWN 19 and DEFUSE-3 20 demonstrated that thrombectomy in selected patients with LVO of the anterior circulation is effective and safe up to 24h after the last time seen well (LTSW). These results showed that a plastered time window for stroke treatment was already outdated.

The DAWN 19 study investigated MT performed within 6–24 hours after stroke, including wake-up strokes, all using a Trevo stent retriever. The inclusion criteria were occlusion of the intracranial ICA or proximal MCA and a mismatch between the clinical deficit and the infarction volume: ≥80yearsgroup, NIHSS≥10 and infarction volume < 21ml; <80yearsgroup, NIHSS≥10 and infarction volume < 31ml or NIHSS≥20 and infarction volume 31-51ml. The infarction volume was evaluated by diffusion-weighted MRI (DWI) or CTP measured using an automated software (RAPID®, iSchemaView). The co-primary outcomes were the mean score for disability on the modified utility-weighted Rankin scale (ranging from 0 [death] to 10 [no symptoms or disability]) and the functional independence (mRs0-2) at 90 days. After 206 patients, study recruitment was halted in the interim analysis. The mean score on the modified utility-weighted Rankin scale was 5.5 in the MT group and 3.4 in the BMT group. The 90-day functional independence was 49% in the MT group and 13% in the BMT group. The intracranial hemorrhage and mortality did not differ significantly between the two groups.

DEFUSE-3 20 randomized patients with proximal MCA or ICA occlusion, infarction size <70ml, and a relationship between the volume of salvageable tissue on the CTP with RAPID software and the infarction volume≥1.8, from 6 to 16h after LTSW. All thrombectomy devices approved by the FDA were allowed. The study was also terminated early for efficacy after 182 patients were randomized. Thrombectomy was associated with a favorable distribution of functional outcomes at 90 days (aOR 3.36; 95% CI, 1.96-5.77; p <0.001) and a higher percentage of patients who were functionally independent (45% MT vs. 17% BMT group, RR, 2.67; 95% CI, 1.60-4.48; p < 0.001). The 90-day mortality was 14% in the MT group and 26% in the BMT group (p = 0.05) with no significant difference for intracranial hemorrhage.

The large effect size of the treatment (NNT = 3) within the 6-24h window in patients selected by advanced neuroimaging (MRI or CTP) opened the door to questions about whether a larger portion of patients should be considered or whether a selection using simpler imaging (NCCT) could be applied. Recently, it has been suggested in an observational study that a relatively normal NCCT in the 6-24h time window may be sufficient to identify patients who may benefit from MT quickly. 40

The recent Efficacy and Safety of Thrombectomy in Stroke with Extended Lesion and Extended Time Window – TENSION trial, 41 randomized 253 patients with AIS and LVO presented with large core (ASPECTS 3-5) using only NCCT to select patients up to 12 hours of symptoms. The study showed a benefit of MT, with a shift in the distribution of mRS for better outcomes (acOR 2.58, 95% CI 1.60–4.15, p = 0.0001), lower mortality (hazard ratio 0.67, 95% CI 0.46–0.98; p = 0.038) and similar rates of SICH (6% and 5% with MT and BMT, respectively). The clinical-radiological mismatch criteria are being tested in an ongoing RCT in Brazil, the Resilient – Extend trial (NCT04256096).

#### *Thrombectomy for basilar occlusion*

The Basilar artery international cooperation study–BASICS 42 - compared MT versus BMT in 300 basilar artery occlusion (BAO) patients within 6h of symptom onset. IV thrombolysis was used in 79% of the patients. MT was started on average 4.4 hours after stroke. There was no significant difference in good functional outcome (mRS ≤3), at 90 days (44.2% MT group vs. 37.7% BMT group, RR 1.18; 95% CI, 0.92-1.50) or in SICH (4.5% MT, 0.7% control, RR 6.9; CI 95%, 0.9-53.0). However, there were several limitations of this study, with almost 30% of eligible patients not recruited suggesting selection bias.

The Endovascular Treatment versus Standard Medical Treatment for Vertebrobasilar Artery Occlusion – BEST trial 43 randomized 131 patients within 8h of symptom onset at 28 centers in China. The high rates of crossover (21.5%) caused the study to be terminated early. In the intention-to-treat analysis, there was no difference in the proportion of patients with mRS≤3 at 90 days but the evaluation per protocol showed significant superiority of MT (44.45 vs. 25.5%; OR 2.90; 95% CI 1.20 − 7.03, p = 0.04).

Two studies conducted in China published in 2022 showed the effectiveness of MT for BAO. In the Basilar Artery Occlusion Chinese Endovascular Trial – BAOCHE, 44 217 patients within 6-24h of LTSW were randomized. The study was stopped in the interim analysis for superiority of thrombectomy, with 46% of the MT group achieving an mRS0-3 in 90 days compared to 24% in the BMT group (aOR 1.81; 95% CI, 1.26-2.60; p < 0.001). The mortality was 31% vs 42%, respectively (aRR 0.75; 95% CI, 0.54-1.04) and SICH was 6% vs 1% (aRR 5.18; 95% CI, 0.64-42.18). The NNT was 4.5.

In addition, the Endovascular Treatment for Acute Basilar Artery Occlusion -ATTENTION 45 study recruited 340 patients with BAO within 12 hours of symptoms. All patients had NIHSS≥10, (median NIHSS of 24). An mRs0-3 was achieved by 46% in the MT group compared with 23% in the BMT group (aRR 2.06; 95% CI, 1.46-2.91, p < 0.001). NNT 4. The 90-day mortality with MT was lower (37% MT vs. 55% BMT, aRR, 0.66; 95% CI, 0.52-0.82).

#### *Interaction with tPA*

All pivotal studies compared MT with BMT, which included IV thrombolysis in all eligible patients (70% of cases). Because of this, current international recommendations advocate that thrombolysis should be offered to all patients eligible for this treatment before MT. 5 46

In favor of tPA, we have the possibility of early reperfusion because of the ability of thrombolysis to be initiated more quickly than thrombectomy. A meta-analysis found complete recanalization within 3h of initiation of IV tPA in 21% of M1 occlusions, 38% of M2, and 4% of ICA. 47 Early recanalization was also observed in the main studies of MT, 14 15 16 17 18 due to the number of patients referred in the drip-and-ship model (transport to a comprehensive stroke center while receiving IV thrombolysis). Other reasons that favor the administration of tPA before MT are its theoretical function of "softening" the thrombus and facilitating the endovascular procedure, making it necessary a smaller number of passages, shorter procedure times, lower costs, and higher rates of successful reperfusion; chance of reperfusion even in failed procedures or thrombi inaccessible by technical adversities or anatomical; possibility of reperfusion of distal branches and microcirculation, which are not reached by currently available devices. The arguments against the administration of tPA before MT are its low effectiveness rates in most patients with LVO with high thrombotic load, 48 higher risks of bleeding (intracranial, systemic, and at the access site), which may even outweigh the benefits in populations with the set of certain risk factors such as advanced age, extensive strokes, hyperglycemia, chronic kidney diseases, leukoaraiosis and microbleeding, 29 49 impediment of the interventionist who performs the procedure in using other antithrombotic drugs (sometimes necessary) as heparin, glycoprotein IIb/IIIa receptor antagonists and other antiplatelet agents; risk of distal embolization (to an inaccessible branch) of the thrombus due to friability caused by thrombolytic 50 ; and the costs added to the treatment.

Six RCTs have attempted to prove the non-inferiority of direct MT (without IV thrombolysis in eligible patients) compared to combined treatment (thrombolysis plus thrombectomy). In the metanalysis of individual patients 51 from these six studies (n = 2313) the median mRS score at 90 days was 3 (IQR 1-5) for MT alone and 2 (1-4) for combined therapy (acOR 0.89, 95%CI 0.76-1.04). SICH and mortality rates did not differ significantly. Some studies showed non-inferiority, and others did not. The metanalysis did not achieve non-inferiority of MT alone compared to combined treatment in patients presenting directly at endovascular treatment centers.

Some important points to be emphasized are the fact that all the studies cited were conducted in centers of excellence, with well-established protocols and pathways of care, had interventional neuroradiologists and hemodynamic rooms immediately available on-site and only include patients who arrived directly at the advanced center. All these characteristics make the results of direct thrombectomy groups very little reproducible, especially in LMIC countries, such as Brazil. Therefore, it is still recommended to offer thrombolytic treatment to all eligible patients and to individualize the indications for direct thrombectomy based on clinical and imaging characteristics, as well as the availability of professionals and equipment. We are waiting for the results of RESILIENT Direct–TNK (study enrolling patients in Brazil) to answer this open question (NCT05199194).

#### STROKE IN BRAZIL

Acute stroke care has improved over the last twenty-five years in Brazil. In 1996, the Brazilian Stroke Society was founded with the aim of guiding stroke care in the country. The first stroke unit in Brazil was created in 1997, in Joinville, and only four years later, in 2001, tPA therapy for AIS was approved by the National Regulatory Agency (ANVISA). At that time, private hospitals began to implement IV thrombolysis for stroke, and a few years later, some university public hospitals started to implement it with local financial resources. In 2008, after the good results of the implementation of 35 stroke services in the country, including 15 public services, the Ministry of Health (MoH) started a pilot project together with the Brazilian Stroke Network (BSN), a non-governmental organization founded in the same year by stroke neurologists, to create and implement a national stroke plan. From 2008 to 2009, 20 of the 26 Brazilian states were visited and meetings were held with local health authorities, implementing stroke services with local resources linked to the National pre-hospital Emergency Medical Service (SAMU). 2

Finally, in 2012, after the good results of the pilot project, the National Stroke Policy was published by the Brazilian MoH, with the approval of tPA in public health system, the creation of the Acute Stroke Services and the approval of Stroke Care Pathway, which reinforces the importance of organizing the entire network in each region, and not just implementing acute stroke treatment1. 2 8 9 10 The main actions were the definition of requirements and levels of stroke centers; increased funding for stroke care in stroke units and training of healthcare professionals at all levels.

The number of stroke centers in Brazil has increased since then, from 35 stroke centers in 2008 to more than 300 in 2023, 100 of them licensed and sponsored by the MoH.

However, 77% of the stroke centers are in the South and Southeast regions. To decrease disparities and increase access to specialists and thrombolytic treatment, a Telestroke program was created in 2019 using a mobile smartphone tool (Join App ^®^ ), used to help manage acute stroke in real-time and decide the best treatment for each patient. This is a public-private partnership (Brazilian Stroke Network, Allm Inc., and Angels Initiative) that currently serves 35 public hospitals in the country. In three years, 3012 cases were discussed 1027 stroke patients were treated with IV thrombolysis through this Telestroke program, with good results. 52

The lack of evidence of MT efficacy for strokes with LVO in LMIC led the Brazilian MoH to sponsor the Resilient trial in twelve public hospitals, from 2017 to 2020, to evaluate the effectiveness and cost-effectiveness of MT in Brazil. The results showed that the overwhelming efficacy and cost-effectiveness of thrombectomy persists despite the limitations of the public healthcare system. 38 After the trial results MoH approved the MT treatment on February 2021, 11 and the stroke centers are currently awaiting promulgation of the final ordinance for its effective implementation. In 2021 Brazil joined the World Stroke Organization Certification of Stroke Centers Program with in-person assessment, action that has improved the quality of stroke care in the country.

**Figure 5:**
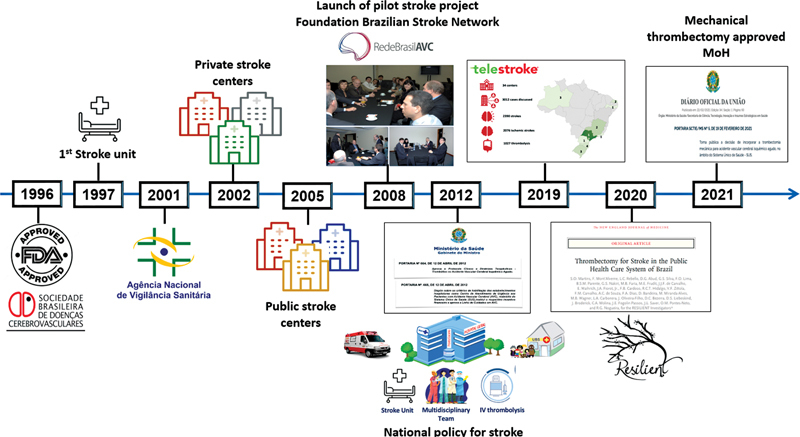
Main milestones in the implementation of reperfusion therapy in Brazil.

Figure 5 shows the main milestones in the implementation of reperfusion therapy in Brazil.
